# Transaminase and Creatine Kinase Ratios for Differentiating Delayed Acetaminophen Overdose from Rhabdomyolysis

**DOI:** 10.5811/westjem.2018.3.37076

**Published:** 2018-06-29

**Authors:** Joshua B. Radke, Douglas A. Algren, James A. Chenoweth, Kelly P. Owen, Jonathan B. Ford, Timothy E. Albertson, Mark E. Sutter

**Affiliations:** * University of Iowa Hospitals and Clinics, Department of Emergency Medicine, Iowa City, Iowa; † Truman Medical Center, Department of Emergency Medicine, Kansas City, Missouri; ‡ University of Missouri-Kansas City, Department of Emergency Medicine, Kansas City, Missouri; § University of California, Davis, Department of Emergency Medicine, Sacramento, California; ¶ University of California, Davis, Department of Internal Medicine, Sacramento, California; || Veterans Administration Northern California Health Care System, Department of Medicine, Mather, California

## Abstract

**Introduction:**

Rhabdomyolysis and delayed acetaminophen hepatotoxicity may be associated with elevated serum transaminase values. Establishing the cause of elevated transaminases may be especially difficult because of limited or inaccurate histories of acetaminophen ingestion. We hypothesized that the comparative ratios of aspartate aminotransferase (AST), alanine aminotransferase (ALT), and creatine kinase (CK) can differentiate acetaminophen hepatotoxicity from rhabdomyolysis.

**Methods:**

A retrospective chart review of patients in four hospitals from 2006 to 2011 with a discharge diagnosis of acetaminophen toxicity or rhabdomyolysis was performed. Subjects were classified into three groups: rhabdomyolysis, acetaminophen overdose (all), and acetaminophen overdose with undetectable serum acetaminophen concentrations [acetaminophen(delayed)]. Ratios of AST, ALT, and CK were compared using non-parametric statistical methods.

**Results:**

1,353 subjects were identified and after applying our exclusion criteria there were 160 in the rhabdomyolysis group, 68 in the acetaminophen overdose (all) group, and 29 in the acetaminophen (delayed) group. The AST/ALT ratio for the rhabdomyolysis group was 1.66 (Interquartile range: 1.18–2.22), for the acetaminophen overdose (all) group was 1.38 (1.08–1.69, statistically lower than the rhabdomyolysis group, p = 0.018), and for the acetaminophen (delayed)group was 1.30 (1.06–1.63, p = 0.037). CK/AST ratios were 21.3 (12.8–42.2), 5.49 (2.52–15.1, p < 0.001), and 3.80 (1.43–13.8, p < 0.001) respectively. CK/ALT ratios were 37.1 (16.1–80.0), 5.77 (2.79–25.2, p < 0.001), and 5.03 (2.20–17.4, p < 0.001) respectively. Increasing CK to transaminase ratio cutoffs resulted in increasing test sensitivity but lower specificity.

**Conclusion:**

AST/ALT, CK/AST and CK/ALT ratios are significantly larger in rhabdomyolysis when compared to patients with acetaminophen toxicity. This result suggests that the ratios could be used to identify patients with rhabdomyolysis who otherwise might have been diagnosed as delayed acetaminophen toxicity. Such patients may not require treatment with N-acetylcysteine, resulting in cost savings and improved resource utilization.

## INTRODUCTION

Differentiating delayed presentations of acetaminophen toxicity from rhabdomyolysis can be difficult for many reasons. First, both rhabdomyolysis and acetaminophen toxicity can be associated with increased aspartate aminotransferase (AST) and alanine aminotransferase (ALT) values.^1^,^2^ Second, though patients may go on to develop hepatotoxicity from acetaminophen, their serum acetaminophen concentrations may be low or undetectable because of a delay between ingestion and hospital presentation. Lastly, patients with these conditions can be found in an unconscious state and may be able to provide little or no clinical history. Medical toxicologists and poison centers are frequently consulted with the question of whether the transaminase elevations could be due to delayed acetaminophen toxicity and if treatment with N-acetylcysteine (NAC) is required.

No common clinical tool or laboratory test can be used to help differentiate the transaminase elevation of acetaminophen toxicity from that of rhabdomyolysis. Investigators have evaluated measurement of gamma-glutamyltransferase, isoforms of ALT, and acetaminophen adducts for this purpose, but these are impractical or not routinely available in clinical practice.^3^–^6^ Many physicians rely on their clinical experience in differentiating between these two conditions, whereas others treat all patients with possible acetaminophen toxicity with NAC in order to avoid possible hepatic injury.^2^ Given the lack of specific findings on examination or data-driven guidance from the literature, both practices are reasonable.

Since there are no objective measures to determine whether a transaminase elevation is from a delayed acetaminophen overdose or from rhabdomyolysis, we sought to determine if the relative values of ALT, AST and creatine kinase (CK) could be used for this purpose. We hypothesized that the ratios of AST/ALT, CK/AST, and CK/ALT would be higher in patients with rhabdomyolysis than in patients with acetaminophen toxicity.

## METHODS

We performed a multi-center, retrospective chart review of admitted patients seen at four tertiary care, university hospitals, including one children’s hospital, from January 2006 to October 2011. We obtained electronic medical records (EMR) on all patients with the discharge diagnosis of rhabdomyolysis or acetaminophen overdose. Subjects were included if they had a discharge diagnosis of acetaminophen overdose or rhabdomyolysis and age ≥ 10 years. Data on those patients were extracted from the EMR, de-identified, and then evaluated for inclusion and exclusion criteria by two authors (JR and DA). The data extracted included age, sex, diagnosis, and laboratory values including AST, ALT, and CK. We excluded subjects if laboratory data were incomplete (CK, AST, or ALT values missing), or if the subject was a prisoner or pregnant. Children less than 10 years old were also excluded because the children’s hospital provided care for neuromuscular diseases, inborn errors of metabolism, and other genetic diseases that might have caused transaminase elevations for reasons other than rhabdomyolysis. The concentrations used for CK, AST, and ALT were the values on presentation, as these would likely be the results used to determine whether or not NAC is indicated for possible acetaminophen toxicity.

**Population Health Research Capsule**

What do we already know about this issue?

Aspartate aminotransferase (AST) and alanine aminotransferase (ALT) elevations can be seen in both acetaminophen overdoses and rhabdomyolysis, and differentiating between the two can often be difficult.

What was the research question?

Can commonly obtained labs be used to differentiate between rhabdomyolysis and acetaminophen toxicity?

What was the major finding of the study?

An elevated AST/ALT, creatine kinase (CK)/ALT, or CK/ALT ratio suggests that rhabdomyolysis may be more likely than acetaminophen toxicity.

How does this improve population health?

A rule similar to this may help improve resource utilization for patients with rhabdomyolysis in whom acetaminophen toxicity is unlikely.

We abstracted data from the EMRs and entered them into an Excel spreadsheet (Microsoft Corp, Redmond, Washington, USA). The data were analyzed by the use of R^©^ (The R Foundation for Statistical Computing, version 3.1.1). We categorized the patients into three groups for analysis based on discharge diagnosis (based on ICD-9 coding by hospital coders): rhabdomyolysis, acetaminophen overdose (all), and acetaminophen overdose (delayed). The acetaminophen group was broken into two separate groups because we are most interested in differentiating between cases of rhabdomyolysis and patients with a delayed presentation of acetaminophen overdose with an undetectable acetaminophen level. The acetaminophen (all) group included all patients who had a discharge diagnosis of acetaminophen overdose, regardless of initial acetaminophen concentration. The acetaminophen (delayed) group only included those who had a discharge diagnosis of acetaminophen overdose, but had an undetectable acetaminophen level on admission. The primary outcome measures were the ratios of AST to ALT, CK to AST, and CK to ALT. We tested data normality with the Shapiro-Wilk test. Because all data were non-parametric, we used the Kruskall-Wallis test to determine if there were differences between groups; when differences were present, we performed pairwise testing by use of the Mann-Whitney-Wilcox test with a Bonferroni correction for multiple-hypothesis testing. In all cases, a p-value of <0.05 was considered statistically significant. We also analyzed the sensitivity, specificity, positive likelihood, and negative likelihood ratio of various CK/AST and CK/ALT ratio cutoffs as a test to differentiate between rhabdomyolysis cases and acetaminophen overdose cases (both acute and chronic).

The study was approved by the institutional review board of each participating hospital, with approval for data to be shared and coordinated through the University of California, Davis.

## RESULTS

We identified 1,353 subjects with rhabdomyolysis or acetaminophen overdose. A majority in each group were excluded from statistical analysis due to missing laboratory data needed to calculate one or more of the ratios. Excluding those with incomplete data resulted in 160 in the rhabdomyolysis group, 68 in the acetaminophen overdose (all) group, and 29 in the acetaminophen overdose (delayed) group.

**Figure 1:**
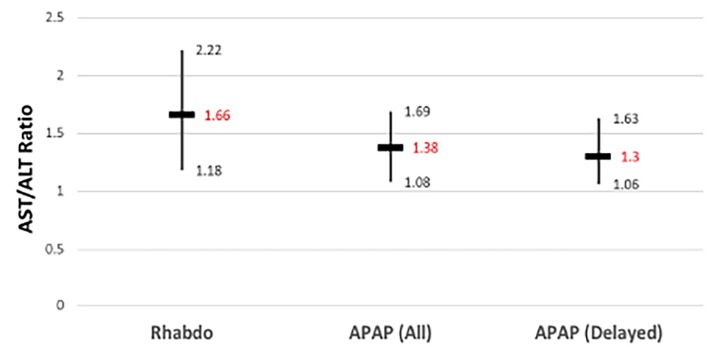
AST/ALT ratios of the three patient groups. *Rhabdo*, rhabdomyolysis group; *APAP (all)*, all patients with acetaminophen overdose; *APAP (delayed)*, patients with delayed acetaminophen toxicity. Vertical bars indicate interquartile range of values; horizontal bars indicate median values.

Patient demographics and the ratios of AST to ALT, CK to AST, and CK to ALT along with the results of the Kruskall-Wallis comparisons are summarized in Table 1. There was a statistically significant difference in the AST/ALT ratio between the rhabdomyolysis group and the two acetaminophen overdose groups (p=0.018 for acetaminophen (all) and p=0.037 for acetaminophen (delayed). There was no difference between the two acetaminophen groups (p=1.00). Medians and interquartile ranges (IQR) are demonstrated in Figure 1.

**Figure 2:**
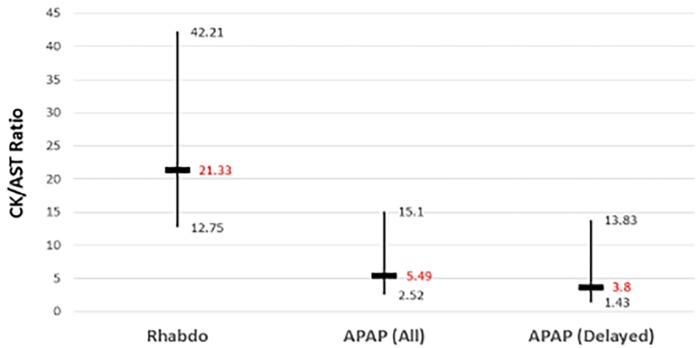
CK/AST ratios of the three patient groups. *AST*, aminotransferase; *CK*, creatine kinase; *Rhabdo*, rhabdomyolysis group; *APAP (all)*, all patients with acetaminophen overdose; *APAP (delayed)*, patients with delayed acetaminophen toxicity. Vertical bars indicate interquartile range of values; horizontal bars indicate median values.

The CK/AST ratios for the rhabdomyolysis, acetaminophen overdose (all), and acetaminophen overdose (delayed) groups were 21.3 (IQR 12.8–42.2), 5.49 (IQR 2.52–15.1), and 3.80 (1.43–13.8) respectively (Figure 2, p<0.001). Pairwise comparisons revealed statistically significant differences between the rhabdomyolysis group and the acetaminophen overdose (all) group (p<0.001), as well as the rhabdomyolysis and the acetaminophen overdose (delayed) group (p<0.001). There was no statistical difference in the CK/AST ratio between the two acetaminophen-overdose groups (p=1.00).

**Figure 3:**
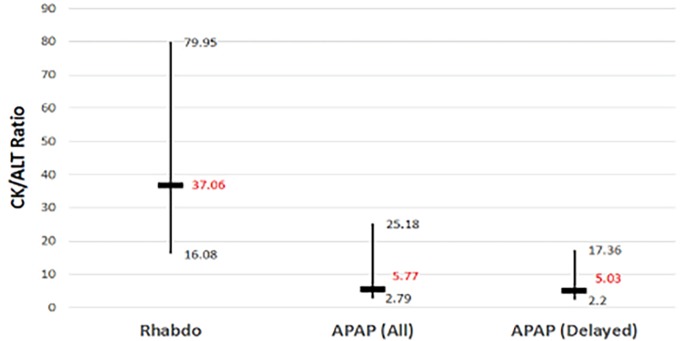
CK/ALT ratios of the three patient groups: rhabdomyolysis group; patients with acetaminophen overdose (all); and those with acetaminophen overdose (delayed). *Rhabdo,* rhabdomyolysis group; *APAP (all)*, all patients with acetaminophen overdose; *APAP (delayed)*, patients with delayed acetaminophen toxicity. Vertical bars indicate interquartile range of values; horizontal bars indicate median values.

The CK/ALT ratios for the rhabdomyolysis, acetaminophen overdose (all), and acetaminophen overdose (delayed) groups were 37.1 (IQR 16.1–80.0), 5.77 (2.79–25.2), and 5.03 (2.20–17.4) respectively (Figure 3, p<0.001). Pairwise comparisons revealed statistically significant differences between the rhabdomyolysis group and the acetaminophen overdose (all) group (p<0.001), as well as the rhabdomyolysis and the acetaminophen overdose (acetaminophen negative) group (p<0.001). There was no statistical difference in the CK/ALT ratio between the two acetaminophen overdose groups (p=1.00).

The test characteristics for different CK-to-transaminase ratios are presented in Table 2 (with ratios below the cutoff designated as positive for acetaminophen overdose). Increasing the ratio cutoff resulted in improved sensitivity but markedly reduced specificity.

## DISCUSSION

Measurements of AST and ALT and their relationship with CK would seem to be useful in helping to differentiate rhabdomyolysis from delayed acetaminophen toxicity. AST and ALT are enzymes that play many vital roles, including amino acid metabolism and gluconeogenesis. 7 ALT is found predominantly in the liver, but is also present in skeletal and heart muscle. AST is found more widely throughout the body including the heart, brain, skeletal muscle, and liver.^8^ Despite the wide distribution of these enzymes, clinically they are used mainly as markers of hepatic injury, when they are thought to “leak out” of damaged cells into the blood.^9^ Since AST and ALT are present throughout the body, they may be elevated in conditions not involving the liver; and indeed both AST and ALT can be elevated in rhabdomyolysis in the absence of liver injury. 10

In the search for the cause of abnormally elevated transaminases, serum CK is often measured. The biological role of CK is that of catalyzing the phosphorylation of creatine, thus producing phosphocreatine. Phosphocreatine, in turn, rapidly produces ATP in tissues that have high energy demand. 11 CK is predominantly located in skeletal muscle, myocardium, and the brain, and serum CK values can be elevated and used as a marker of injury to these organs. 8 Although CK is present in the liver, its concentration in hepatic tissues is significantly lower than in other tissues. 8,12

The results of this study support our hypothesis that the ratios of AST/ALT, CK/AST and CK/ALT would be higher in patients with rhabdomyolysis than in patients with acetaminophen toxicity. This observation may be useful in differentiating patients with rhabdomyolysis from those who otherwise might be considered candidates for treatment with NAC for possible delayed acetaminophen poisoning. Avoiding the unnecessary treatment of patients with NAC, and possibly longer hospital length of stay, could reduce the cost of care.

Additionally, our finding of a higher ratio of CK to ALT than that of CK to AST is consistent with the tissue distribution of these enzymes; that is, higher values for AST than for ALT are more likely in rhabdomyolysis because the concentrations of AST in skeletal muscle are higher than those of ALT. 8

Besides possible cost savings, it is important to minimize risk of exposure to unnecessary medications for our patients. Although NAC is generally safe, anaphylactoid reactions, sometimes resulting in death, have been reported with intravenous (IV) administration.^13^ Deaths have also been attributed to iatrogenic errors with IV formulations of NAC. 13,14 In patients with rhabdomyolysis, taking the risk of an iatrogenic complication from NAC may not be warranted. 15,16

Our data suggest higher CK/AST or CK/ALT ratios are more likely to be seen with rhabdomyolysis than acetaminophen ingestion. This is not to say that the ratio can be used in a vacuum to differentiate patients with rhabdomyolysis from those with acetaminophen toxicity. Instead, it would ideally be used in those patients for whom there is an already-low likelihood of acetaminophen toxicity that cannot be excluded due to a limited history.

## LIMITATIONS

Our study has several limitations. First, it is retrospective, with the inherent shortcomings associated with this study design. Second, patient-selection bias could be present because the majority of the patients in each group were excluded because of missing laboratory data; in the cases of acetaminophen overdose, the CK values often were absent, likely because CK is not a routinely ordered lab test on patients who have suspected overdose. Third, the discharge diagnoses may not have always been accurate, and coding errors may have existed that resulted in patient misclassification. We also did not verify each of the discharge diagnoses. Finally, the values of AST, ALT, and resulting ratios could have been different depending on when they were drawn in the course of a patient’s toxicity, as AST and ALT concentrations rise and fall at different rates.^17^

## CONCLUSION

In summary, we found the AST/ALT, CK/AST, and CK/ALT ratios were significantly higher in patients with rhabdomyolysis than in patients with acetaminophen toxicity. This result suggests that the ratios could be used to identify patients with rhabdomyolysis who otherwise might have been diagnosed as delayed acetaminophen toxicity. Such patients may not require hospitalization and treatment with N-acetylcysteine, resulting in considerable cost savings and decreased resource utilization. Based on the limitations of our study, however, these ratios are not ready for clinical use. Prospective validation of our findings in a diverse patient population is needed before these ratios can be applied in regular clinical practice.

## Figures and Tables

**Table 1:** Demographics and results summary. Ratios are expressed as medians, with interquartile ranges.

	Rhabdo	APAP all	APAP delayed	P value
Age	48 (34–56)	42 (29–52)	46 (37–55)	NA
Male	115 (71.9%)	38 (55.9%)	18 (62%)	NA
AST/ALT	1.66 (1.18–2.22)	1.38 (1.08–1.69)	1.30 (1.06–1.63)	0.003396
CK/AST	21.33 (12.75–42.21)	5.49 (2.52–15.10)	3.80 (1.43–13.83)	<0.001
CK/ALT	37.06 (16.08–79.95)	5.77 (2.79–25.18)	5.03 (2.20–17.36)	<0.001

P values represent the results of the Kruskall-Wallis test of comparison between the rhabdomyolysis group and the acetaminophen (delayed) group.

*APAP*, acetaminophen; *rhabdo*, rhabdomyolysis; *AST,* aspartate aminotransferase*; ALT*, alanine aminotransferase; *CK*, creatine kinase.

**Table 2:** Sensitivity and specificity of different CK* to transaminase ratio cutoffs.

Ratio cutoff	Sensitivity (95% CI)	Specificity (95% CI)	Positive likelihood ratio (95% CI)	Negative likelihood ratio (95% CI)
CK/AST				
15	75.3% (65.5–83.5%)	68.8% (61–75.8%)	2.41 (1.86–3.11)	0.36 (0.25–0.52)
20	83.5% (74.6–90.3%)	53.2% (45.1–61.2%)	1.78 (1.48–2.16)	0.31 (0.19–0.5)
25	84.5% (75.8–91.1%)	43% (35.1–51.1%)	1.48 (1.26–1.74)	0.36 (0.22–0.59)
30	89.7% (81.9–94.9%)	35.3% (27.8–43.3%)	1.39 (1.21–1.58)	0.29 (0.16–0.55)
CK/ALT				
15	67% (56.7–76.2%)	76.9% (69.6–83.2%)	2.9 (2.11–3.97)	0.43 (0.32–0.58)
20	73.2% (63.2–81.7%)	71.8% (64–78.7%)	2.6 (1.97–3.43)	0.37 (0.26–0.53)
25	76.3% (66.6–84.3%)	64.1% (56–71.6%)	2.13 (1.68–2.69)	0.37 (0.25–0.54)
30	83.55 (74.6–90.3%)	59% (50.8–66.8%)	2.04 (1.65–2.51)	0.28 (0.18–0.45)

*CK*, creatine kinase; *AST*, aspartate aminotransferase; *ALT*, alanine aminotransferase; *CI*, confidence interval.

## References

[1] Torres P, Helmstetter J, Kaye A (2015). Rhabdomyolysis: pathogenesis, diagnosis, and treatment. Ochsner J.

[2] Heard K (2008). Acetylcysteine for acetaminophen poisoning. N Engl J Med.

[3] Whitfield J (2001). Gamma glutamyl transferase. Crit Rev Clin Lab Sci.

[4] Pertusi R, Dickerman R, McConathy W (2001). Evaluation of aminotransferase elevations in a bodybuilder using anabolic steroids: hepatitis or rhabdomyolysis. J Am Osteopath Assoc.

[5] Miyazaki M, Resonblum JS, Kasahara Y (2009). Determination of enzymatic source of alanine aminotransferase activity in serum from dogs with liver injury. J Pharmacol Toxicol Methods.

[6] Bond G (2009). Acetaminophen protein adducts: a review. Clin Toxicol.

[7] Walker H, Hall WD, Hurst JW (1990). Creatine Kinase. Clinical Methods: The History, Physical, and Laboratory Examinations.

[8] Dufour D, Lott JA, Nolte FS (2000). Diagnosis and monitoring of hepatic injury. I. Performance characteristics of laboratory tests. Clin Chem.

[9] Ramaiah SA (2007). Toxicologist guide to the diagnostic interpretation of hepatic biochemical parameters. Food Chem Toxicol.

[10] Weibrecht K, Dayno M, Darling C (2010). Liver aminotransferases are elevated with rhabdomyolysis in the absence of significant liver injury. J Med Toxicol.

[11] Wallimann Y, Wyss M, Brdiczka D (1992). Intracellular compartmentation, structure and function of creatine kinase isoenzymes in tissues with high and fluctuating energy demands: the ‘phosphocreatine circuit’ for cellular energy homeostasis. Biochem J.

[12] Joseph J, Cardesa A, Carreras J (1997). Creatine kinase activity and isoenzymes in lung, colon and liver carcinomas. Br J Cancer.

[13] Sandilands E, Bateman D (2009). Adverse reactions associated with acetylcysteine. Clin Toxicol.

[14] Elms A, Owen KP, Albertson TE (2011). Fatal myocardial infarction associated with intravenous N-acetylcysteine error. Int J Emerg Med.

[15] Lameire N, De Vriese A, Vanholder R (2003). Prevention and nondialytic treatment of acute renal failure. Curr Opin Crit Care.

[16] Fernandez-Funez A, Polo FJ, Broseta L (2002). Effects of N-acetylcysteine on myoglobinuric-acute renal failure in rats. Ren Fail.

[17] Curtis R, Sivilotti M (2015). A descriptive analysis of aspartate and alanine aminotransferase rise and fall following acetaminophen overdose. Clin Toxicol.

